# Psychometric properties of the arabic version of the positive/negative experiences of parents for school-aged students about online learning during COVID-19 pandemic assessment scale

**DOI:** 10.1186/s13104-023-06299-x

**Published:** 2023-02-28

**Authors:** Eman Bajamal, Samaa Al Anazi, Ola Esheaba, Neama Hantira

**Affiliations:** 1 College of Nursing, King Saud bin Abdulaziz University for Health Sciences, and King Abdullah International Medical Research Center (KAIMRC), Jeddah, Saudi Arabia; 2 Faculty of Nursing, Alexandria University, Sidi Gaber, Egypt; 3 College of Nursing, King Saud bin Abdulaziz University for Health Sciences, King Abdullah International Medical Research Center (KAIMRC), Mail Code 6565, P.O. Box 9515, Jeddah, 6565 Saudi Arabia

**Keywords:** Saudi Arabia, COVID-19, Online education, Psychometric properties

## Abstract

**Background:**

The current literature examining the impact of online learning on parents and their children, specifically in the time of COVID-19 are still lacking in Saudi Arabia. Therefore, the aim of the study to evaluate the psychometric properties of the Arabic version of the positive/negative experiences of online learning during COVID-19 Pandemic Assessment Scale.

**Methods:**

A cross-sectional study approved by a university institutional review board was conducted among 184 participants in 2021. The scale was translated from English into Arabic and culturally adapted as needed. The psychometric properties of the instruments, including face and content validity, and internal consistency were evaluated. Exploratory factor analysis (EFA) was conducted to cross-validate the factor structure. The Spearman’s Rho Correlation was used to assess convergent validity.

**Results:**

Cronbach’s alpha were 0.890 and 0.892, respectively, indicating acceptable internal consistency. Item-total correlation coefficients ranged from 0.52 to 0.73 and 0.43 to 0.76, respectively. The EFA indicated a single-factor with a total% variance 52.89 for the positive experience items of the scale and 56.83 for the negative experience items of the scale.

**Conclusion:**

The Arabic version of the the positive/negative experiences of online learning during COVID-19 Pandemic Assessment Scale is reliable and valid measure for assessing parents experiences among Arabic-speaking population.

## Introduction

The year of 2020 and the spread of COVID-19 transformed the world in all its aspects of life, in terms of health, social interaction and most importantly education. Countries worldwide created several measures to overcome the spread of the virus including online learning to enforcee social distancing [1].

In Saudi Arabia, the first confirmed case of COVID-19 in February, 2020. Awareness about the nature of the disease, its severity and its mode of transition was not predictable and the concern about the health of the public was rising. Therefore, the governemnt enforced the practice of social distencing as a way to combat the spread of the virus, especially among vulenrable populations such as school aged childern. As a results of all of these combined factors, the education transferred to online and learning entered a trnasformative stage like never before in the history of mankind [2].

The quick transformation of education from being physical to virtual introduced multiple advantages and disadvatnges to both childern and parents. Parents had to adapt for their childern a new way of learning to ensure continuety of education and maintain acadamic achievment, yet struggle with everyday demanding role they already undertake [3].

Due to the gravity of COVID-19 and its devastative consequences, many parents agree with school closure. Nonetheless, parents face challenges of online learning such as inability to manage multiple responsibilities, inability to motivate children to online learning and the uncertainty of the effectiveness of online learning [4].

Body of knowledge and reaserch have been affected as well with the emergencee of COVID-19, and the impact of the pandamic on parental experience with online learning need to be exmined. To truley examine parental experince and explore the scales used to capture this phenomna, especially among Arabic speaking populations in KSA is crucial to be validated and proved to be reliable.

Due to the unprecedented transformation of education to online learning during the pandemic, there was not many literatures aim to examine parental experiences towards online learning. There are multiple studies aim to address parents’ perception towards the COVID-19, such as Spinelli, Lionetti, Pastore, and Fasolo (2020) [5], Fontanesi, et al. (2020) [6], and Apriyanti (2020) [7]. The authors chose to adopt Garbe et al. (2020) who explored parental experiences with online learning and created a tool that examined multiple concepts of positive and negative experiences towards online learning, faced challenges and struggles [4]. Tool was translated to Arabic language because it contains concepts that are crucial for the Saudi population and filled the gap in literature. The scale surveyed parents named facilitators and barriers to online education. It also explored parental struggles and its impact on their children. In addition, the tool examined concepts that are needed for online learning to succeed, such as school support, prioritization of subjects, and activities done during the pandemic.

The aim of this paper is to psychometrically analyze an Arabic-language scale that is aimed to explore parental experience towards online learning among Arabic speaking populations living in KSA.

## Methods

### Design, participants, and setting

The convenience sample for this cross-sectional study included 184 participants who requited virtually and completed the survey during the summer 2021. Participants were included in the study if they: (a) a mother or father of a school aged children taking online course, (b) resident in Saudi Arabia, (c) speak and read Arabic, and (d) above 18 years old. Participants filled the electronic informed consent prior to answering the questionnaire. The exclusion criteria include a non-parent participant, inability to speak or read Arabic, and younger than 18 years old. Approval from a King Abdullah International Medical Research Center (KAIMRC) in Saudi Arabia were obtained before any data collection.

### Procedures

To capture parental experiences and the struggles they face during the school closure due to COVID-19 pandemic, Garbe et al. (2020) developed an online survey aimed to collect data about the variable understudy. The survey targeted parents to capture their opinion about the school closure [4]. Garbe et al. (2020) established reliabilty of the qualitiatve data tool by employing a fourth researcher who conducted a blind review of the data by assigning codes that was already written by the previous researcher to the existing data. As a result interrater reliability was established with a score of 76% [4].

#### Translation procedure

Measurement of parental sociodemographic, experiences with online learning, and utilized adaptation strategies was adopted from Garbe et al. (2020) [4], and then the tool was translated to Arabic language. The translation was conducted using the four steps recommended by Furr and Bacharach (2008) [8] and Wlatz et al. (2010) [9], which include: forward translation, revision of translation, back-translation, and revision of back-translation. The tool was translated to Arabic by a bilingual native Arabic translator who is fluent in English in a certified translation center in Saudi Arabia. After that, the tool was translated back to English by another translator who is fluent in English in another certified translation center in Saudi Arabia. Then, the English, Arabic and back-translated versions of the tool were compared.

#### Face validity

The scale was assessed for its face validity by a total of *N* = 10 Arabic-speaking individuals in Jeddah, Saudi Arabia: five Arabic-speaking females or other women with a graduate level of education, and five Arabic-speaking males with higher levels of education. Concurrent and retrospective interviewing were utilized to confirm the comprehensibility and simplicity of the survey. In concurrent interviewing, the participants were asked to think out loudly as they fill out the survey to help the researchers gain insight into the clarity of the scale. As in retrospective interviewing, a probing question was provided to respondents after they fill out the survey [10].The purpose of this procedure is to ensure that the survey is easily comprehensible and that the flow of the questions is not confusing for the participants.

Due to discrepancy between the original study Garbe et al. (2020) who investigated parental experiences in an English population with difference of cultural and religious background [4], Modification of the Arabic tool was made to accommodate the Arabic population understudy. The items added demographics were the parents responsible for supervising the online learning, their background in education, knowledge in the use of technology. In addition, since this study was done a year after the original study was published, an item was added to explore parental stress after a year-long of online learning. The Arabic version of the tool utilized the main concepts that were discovered in the results by Garbe et al. (2020) who identified these concepts via qualitative data [4]. Then, the modified Arabic tool used these concepts and transfer it to be measured quantitatively.

The Arabic version examined detailed aspects of the negative experiences towards online learning, such as student interaction with their teacher, loss of academic motivation, social interaction with peers, sense of boredom, impact of online learning on child normal development, along with physical disadvantages. The positive experiences were detailed as well, such as child utilization of educational resources, sense of comfort being at home, enhancement of child technological skills, bonding with family members and identification of child weak points in learning. The needed modification and adjustments to the survey based on the results of retrospective and concurrent interviews were done.

The Arabic version of the tool consists of 16 demographic questions, followed by 8 questions aim to assess parental percetion and attitudes towards online learning, all the question were close ended. The parental experinces towards online learning utilized 5 point likert scale, starting with negative experinces with 12 items, then the positive experinces was measured with 11 items. All the experiences items had scores ranging from 1 to 5 (lowest to highest). The scoring criteria were as follows: 1–2 indicated low score of the construct, 2–3 indicated moderately low score of the construct, 3–4 indicated moderately high score of the construct, and 4–5 indicated high score of the construct.

#### Content validity

The 4-point ordinal rating relevance scale (1 = not relevant, 2 = somewhat relevant, 3 = quite relevant, and 4 = very relevant was used in this study to evaluate the relevance of the scale’s items [9] by five bilingual Arabic-English speaker experts with expertise in survey development evaluated the survey item for appropriateness and relevance to the theoretical constructs. The content validity of the scales was evaluated using the interrater reliability or agreement, the content validity index (CVI), and the average congruency percentage (ACP) [9, 11]. An instrument with an inter-rater reliability greater than 75% [12, 13], an I-CVI of 0.83, a S-CVI of 0.80 or above [14], and an ACP of 90% or higher is considered acceptable [11].

### Statistical analysis

The Statistical Package for the Social Sciences computer software (SPSS for Mac, Version 23.0; IBM, 2015) was used to analyze data. Descriptive statistics were calculated including: means, standard deviations, and percentages, to describe participant demographics and the study variables. For content validity evaluation, the interrater reliability (or agreement), the CVI, and the ACP were calculated [9, 11]. For construct validity, the EFA was calculated with oblique rotation. For the convergent validity, the Spearman Rho Correlation was calculated since the data was not normally distributed. For internal consistency, Cronbach’s alpha and item-total correlation was calculated [15, 16]. Statistical significance was based on the standard alpha level of 0.05.

## Results

### Demographics characteristics

A total of 184 participants were requited virtually and completed the survey. Around 75.5% of the participants were mothers. More than half of the participants (*n* = 98, 53.3%) were between the age group of 30–40 years. Around 59.8% of the participants had undergraduate degree. For the family income, most of the participants (*n* = 108, 58.7%) were from families who have a family income more than SR12,000. Most of the participants who followed up with their kids for the online learning during COVID-19 were mothers only 121 (65.8%). Table 1 shows the demographic characteristics of the study sample.

**Table 1:** *Sample Demographic Characteristics (N = 184)*

Demographic variable	*n*	%
The person who completes the survey		
Mother	139	75.5
Father	40	21.8
Non-Parent	5	2.70
Age		
< 30 years	7	3.80
30–40 years	98	53.3
40–50 years	64	34.8
> 50 years	15	8.20
Educational level		
Primary school	5	2.7
Intermediate school	2	1.1
High school	23	12.5
Undergraduate degree	110	59.8
Postgraduate degree	44	23.9
Family income		
< SR3,000	8	4.30
SR3,000-SR5,000	10	5.40
SR5,000-SR8,000	25	13.6
SR8,000-SR12,000	33	18.0
> SR12,000	108	58.7
Residency status		
Family house	48	26.1
Separate apartment	136	73.9
Who follow learning remotely		
Mother only	121	65.8
Father only	4	2.20
Mother/father together	53	28.8
Another family member	6	3.30

### Psychometric properties

#### Reliability

As demonstrated in Table 2, the Cronbach’s alpha coefficient was 0.890 with 95% confidence interval for the 11-items positive effects of online learning assessment scale, with item-total correlation coefficients ranging from 0.52 to 0.73 (*α* = 0.890). In addition, as demonstrated in Table 3, the Cronbach’s alpha coefficient was 0.892 with 95% confidence interval for the 12-items negative effects of online learning assessment scale, with item-total correlation coefficients ranging from 0.43 to 0.76 (*α* = 0.892).

**Table 2:** Reliability, exploratory factor analysis of the positive experiences of online learning during COVID-19 Pandemic Assessment Scale

Positive experiences	*M*	*SD*	Item-Total Correlation	Cronbach’s Alpha if Item Deleted
Development child’s research/learning skills	3.5943	0.92287	0.539	0.885
Availability of many educational resources	3.5200	0.86993	0.527	0.886
Feeling comfortable to be at home	3.5600	1.05351	0.605	0.881
Enhancement child’s skills to deal with technology	4.0000	0.89056	0.642	0.879
Enhancement child’s independence skills	3.6286	1.07974	0.577	0.884
Available time for hobbies	3.0743	0.96500	0.623	0.880
Strengthening the social relationship between family members	3.4057	0.98318	0.694	0.876
Strengthen child’s ability to use many educational resources	3.6514	0.92145	0.731	0.874
Identifying the child’s learning strengths/ weakness	3.5029	0.98187	0.563	0.884
Strengthening the relationship with the child on personal and educational level	3.4800	0.95195	0.708	0.875
Be as a teacher for my children as needed	3.5029	0.88327	0.542	0.885
Total % of variance explained/Eigenvalues	52.89			
Cronbach’s alpha of total scale/subscales	0.890			
KMO/Bartlett’s Test of Sphericity	*KMO*: 0.891 *P*: 0.000*			

*KMO: Kaiser-Meyer-Olkin*

*P: P value of KMO/Bartlett’s test*

**Table 3:** Reliability, exploratory factor analysis of the negative experiences of online learning during COVID-19 Pandemic Assessment Scale

Negative experiences	*M*	*SD*	Item-Total Correlation	Cronbach’s Alpha if Item Deleted
Weak direct interaction between teacher & student	4.2429	0.86784	0.433	0.891
Loss of educational motivation and low spirit of competition	4.2429	0.89998	0.607	0.883
Lack of social spirit and loss of ability to build new friendships	4.4915	0.75467	0.448	0.890
Boring from sitting long time in front of PC/Laptop	4.5876	0.70264	0.629	0.883
Child’s forgetfulness/inability to focus/slowness in development of speech/language	4.2090	0.92707	0.630	0.882
Teacher’s inability to assess student performance and achievement	3.9040	1.01513	0.564	0.885
Weak vision due to sitting in front of PC/Laptop	4.1808	0.92998	0.645	0.881
Muscle pain due to sitting in front of PC/Laptop	4.1582	0.93411	0.662	0.880
Weight gain due to lack of movement	4.0678	1.04225	0.586	0.884
Lethargy/headache	4.0282	0.95602	0.760	0.874
Spinal curvature	3.9548	1.01028	0.648	0.880
Indigestion due to stress	3.4011	1.04588	0.616	0.883
Total % of variance explained/Eigenvalues	56.83			
Cronbach’s alpha of total scale/subscales	0.892			
KMO/Bartlett’s Test of Sphericity	*KMO*: 0.873 *P*: 0.000*			

*KMO: Kaiser-Meyer-Olkin*

*P: P value of KMO/Bartlett’s test*

**Content validity.** As shown in Table 4, the interrater reliability coefficients is 93.84 with an acceptable item content validity indexes (I-CVI) as 1.00. The average congruency percentages (ACP) is 95.84 As demonstrated in Table 4, the scale had acceptable content validity based on the three indicators [11–14].

**Table 4:** *Content Validity for the Scale*

Inter-rater reliability coefficient (%)	I-CVI	S-CVI/UA	ACP (%)
93.84	1.00	1.00	95.84

#### Construct validity

An EFA was performed on all the dataset among 184 participants. First, principal component analysis was performed, including all 11 items of the positive effects of online learning during COVID-19 pandemic assessment scale, using a cut-off point of 0.4 for factor loadings as the threshold. Data were suitable for EFA as KMO was greater than 0.5 (0.891) and Bartlett’s test of sphericity was significant (< 0.001). The variance percentage yielded eigenvalues 52.89%. The reliability of the scale was good (*α* = 0.890) which means that the scale is 89% reliable; there is no need to delete any items of the scale. Table 2 shows the EFA of the positive effect scale.

EFA and reliability tests were repeated on the remaining 12 items of the scale. Factor loadings of the items on each item are shown in Table 3. Moreover, an EFA was performed also on all the dataset among 184 participants. First, principal component analysis was performed, including all 12 items of the negative effects of online learning during COVID-19 pandemic assessment scale, using a cut-off point of 0.4 for factor loadings as the threshold. Data were suitable for EFA as KMO was greater than 0.5 (0.892) and Bartlett’s test of sphericity was significant (< 0.001). The variance percentage yielded eigenvalues 56.83%. The reliability of the scale was good (*α* = 0.892) which means that the scale is 89.2% reliable; there is no need to delete any items of the scale. Factor loadings of the items on each item are shown in Table 3.

#### Convergent validity

Based on the correlation matrix among the positive and negative item scores (including 11 and 12 items) of the studied parent’s experiences, all items were significantly and negatively correlated with each other (*r* = -.322, *p* < .01). In other words, those who have negative experiences didn’t have positive ones, and the relationship between them is reversed, and an increase in positive will decrease a negative experience, and vice versa. See Table 5.

**Table 5:** *Spearman’s Rho Correlations Between positive experiences and negative experiences (N = 184)*

Variables	1	2
1- Negative experiences during online learning	—	
2- Positive experience during online learning	− 0.322**	—

Note: *p < .05, **p < .01

The Spearman’s Rho Correlation was used to test convergent validity of the negative experiences during COVID-19 scale. As shown in Table 6, the 11-item of the positive experiences during COVID-19 scale was moderately to strongly correlated [17] with each other that ranging from (*r* = .200, *p* < .01) to (*r* = .674, *p* < .01). In addition, as shown in Table 7, the 12-item of the scale was moderately to strongly correlated [17] with each other that ranging from (*r* = .282, *p* < .01) to (*r* = .648, *p* < .01).

**Table 6:** *Spearman’s Rho Correlations Between positive experiences scale items (N = 184)*

Variables	1	2	3	4	5	6	7	8	9	10	11
1- Development child’s research/learning skills	---										
2- Availability of many educational resources	0.493^**^	---									
3- Feeling comfortable to be at home	0.325^**^	0.312^**^	---								
4- Enhancement child’s skills to deal with technology	0.469^**^	0.417^**^	0.527^**^	---							
5- Enhancement child’s independence skills	0.377^**^	0.339^**^	0.355^**^	0.558^**^	---						
6- Available time for hobbies	0.365^**^	0.423^**^	0.372^**^	0.372^**^	0.388^**^	---					
7- Strengthening the social relationship between family members	0.297^**^	0.325^**^	0.498^**^	0.434^**^	0.431^**^	0.560^**^	---				
8- Strengthen child’s ability to use many educational resources	0.463^**^	0.529^**^	0.464^**^	0.521^**^	0.549^**^	0.539^**^	0.587^**^	---			
9- Identifying the child’s learning strengths/ weakness	0.345^**^	0.231^**^	0.410^**^	0.285^**^	0.306^**^	0.379^**^	0.434^**^	0.353^**^	---		
10-Strengthening the relationship with the child on personal and educational level	0.341^**^	0.310^**^	0.485^**^	0.402^**^	0.439^**^	0.437^**^	0.621^**^	0.518^**^	0.674^**^	---	
11- Be as a teacher for my kids as needed	0.300^**^	0.293^**^	0.400^**^	0.200^**^	0.250^**^	0.345^**^	0.481^**^	0.377^**^	0.418^**^	0.514^**^	---

Note: *p < .05, **p < .01

**Table 7:** *Spearman’s Rho Correlations Between negative experiences scale items (N = 184)*

Variables	1	2	3	4	5	6	7	8	9	10	11	12
1-Weak direct interaction between teacher & student	---											
2- Loss of educational motivation and low spirit of competition	0.631^**^	---										
3- Lack of social spirit and loss of ability to build new friendships	0.467^**^	0.597^**^	---									
4- Boring from sitting long time in front of PC/Laptop	0.356^**^	0.549^**^	0.492^**^	---								
5- Child’s forgetfulness/inability to focus/slowness in development of speech/language	0.495^**^	0.555^**^	0.351^**^	0.554^**^	---							
6- Teacher’s inability to assess student performance and achievement	0.452^**^	0.548^**^	0.324^**^	0.327^**^	0.541^**^	---						
7- Weak vision due to sitting in front of PC/Laptop	0.252^**^	0.282^**^	0.310^**^	0.442^**^	0.526^**^	0.334^**^	---					
8- Muscle pain due to sitting in front of PC/Laptop	0.132	0.307^**^	0.282^**^	0.432^**^	0.321^**^	0.293^**^	0.629^**^	---				
9- Weight gain due to lack of movement	0.122	0.233^**^	0.279^**^	0.437^**^	0.335^**^	0.300^**^	0.477^**^	0.577^**^	---			
10- Lethargy/headache	0.229^**^	0.395^**^	0.299^**^	0.490^**^	0.509^**^	0.418^**^	0.687^**^	0.631^**^	0.575^**^	---		
11- Spinal curvature	0.139	0.317^**^	0.292^**^	0.403^**^	0.312^**^	0.328^**^	0.514^**^	0.593^**^	0.472^**^	0.648^**^	---	
12- Indigestion due to stress	0.128	0.317^**^	0.262^**^	0.295^**^	0.349^**^	0.350^**^	0.501^**^	0.493^**^	0.521^**^	0.593^**^	0.630^**^	---

Note: *p < .05, **p < .01

## Discussion

The COVID-19 pandemic forced the closure of all schools in March 2020 and the start of distance learning from home [18]. This sudden interruption in classroom learning was the most common technique used by governments to combat COVID-19 transmission. Regardless of how important teachers are in children’s education, the current environment prevents them from doing so successfully, and parents are being pushed to fill the gap. On the other hand, parents and family were not well prepared to assume this role [19].

The Arabic version of the tool might have measured the same concepts in the original article, but due to major differences of the school system between the United States and Saudi Arabia. Reconducting the study is crucial to identify any differences between parents from different cultural backgrounds and their experiences towards online learning.

When comparing the results of this study and Garbe et al. (2020) results, both population understudy reported challenges and obstacles towards online learning [4]. Discrepancy in the results of both articles were present, in the Arabic version the most reported negative aspect of online learning is boredom versus Garbe et al. (2020) who reported balancing responsibilities and lack of learner motivation [4]. In regard to the positive experiences, this study participant’s reported high level of enhancement of technological skills among children which was not apparent in the original article.

This study has some strengths that merit highlighting. First, this is the first validated questionnaire that measures parental experience towards online learning among Arabic-speaking populations. It is worthy to note that around three-quarters of the participants were mothers which indicates that these women have multiple tasks to do during the challenging era of the COVID-19 pandemic and online learning. Most of the participants who followed up with their kids for the online learning during COVID-19 were mothers while the rest of the participants were mothers/fathers together compared to the minorities of fathers and other family members. Furthermore, around 60% of the participants had an undergraduate degree. These findings confirm the importance of assessment of the parent’s experience with online learning and its requirements. Parents’ experience with online learning assessment tools was examined to explore its psychometric properties including reliability and validity, especially among Arabic speaking. This procedure will help to appraise the current tool as a scale to assess parents’ experience in upcoming future research.

The current study examined two main important domains in parents’ experience with online learning. These domains include the positive and negative effects of the COVID-19 pandemic on the online learning process. In this regard, tool reliability was tested, the goal of reliability testing is to ensure that tool consistently provides the same result over time [20]. The 11-items positive effects of the online learning assessment scale is 89% reliable and the 12-items negative effects of the online learning assessment scale is 89.2% reliable. The same findings were reported by Hernández-Padilla et al. (2020) who assess the COVID-19- self-efficacy scale (SES) psychometric properties and found that the COVID-19-SES reliability was an exceptionally reliable [21].

The scale demonstrated acceptable content validity based on the three indicators in the current investigation [22–24], with an interrater reliability coefficient of 93.84 and an acceptable I-CVI, with an average congruency percentage of 95.84 [11–14]. These findings are congruent with Hernández-Padilla et al. (2020) who reported that the COVID-19-SES scale’s content validity index is 0.92 [21].

Construct validity is demonstrated experimentally when a survey distinguishes between people who have and don’t have specific qualities. Construct validity should show that a test’s results predict the theoretical attribute it claims to predict [25]. All score-based inferences are supported by the construct validity of score interpretation. The cornerstone of unified validity is that the appropriateness, meaningfulness, and utility of scored-based inferences are all considered; and that this integration is the result of empirically based score interpretation [26].

Moreover, psychometric analysis of the factor analysis is a statistical technique that examines the relationships between a collection of survey questions to see if a participant’s replies to different subsets of items are more closely related. EFA can be used to look for patterns in a batch of data. As a result, EFA can help establish new theories by elucidating how diverse things and constructs interact with one another. During the preliminary stages of instrument development, EFA is appropriate. The researcher can use EFA to identify items in the survey that do not empirically belong to the intended construct and should be deleted. EFA can also be used to investigate the instrument’s dimensionality [27].

The findings of EFA for current study are acceptable based on Wang et al. (2017) who documented that the principal component analysis (PCA) with varimax rotation was used to accomplish EFA [28]. The possibility of doing factor analysis was tested using Bartlett’s test of sphericity and the Kaiser–Meyer–Olkin (KMO) statistic. The eigenvalues of the factors with eigenvalues greater than one were extracted. Factor loadings greater than 0.50 were deemed acceptable.

The findings of convergent validity for current study are supported by Abma et al. (2016) who reported that one sort of validity that is routinely tested for participant-reported outcome measures is convergent validity (PROMs) [29]. It is evaluated using “hypothesis testing,“ which involves examining if the results of the instrument under research correspond with other instruments in an expected way.

To sum up, the psychometric properties of the scale were satisfactory, and the tool can be used to assess either positive or negative effects of COVID-19 on the online learning experience of the Arabic speaking.

## Limitations

Further research is still needed to test all tools used to assess the effects of COVID-19 on the parents, especially mothers’ and family’s experience with online learning as well as its effects from the teacher’s perspective also. Moreover, the study was observational and conducted at one-time point. Testing-retesting did not occur. A cause-effect relationship cannot be determined. A limitation of this study is that did not determine the criterion validity due to the small sample size and future analysis of psychometric properties, such as Confirmatory Factor Analysis (CFA) with a big sample size among Saudi populations.

## Conclusion

Following a comprehensive assessment, the parent’s experience with online learning in the COVID-19 pandemic assessment tool for Arabic speaking has shown to have good psychometric properties. The results suggest that the study scale that measures the positive and negative effects of the COVID-19 pandemic on online learning is a valid, reliable, and legible instrument.

## Data Availability

The datasets used and/or analyzed during the current study are available from the corresponding author on reasonable request.
